# Performance of Circular Concrete-Filled FRP-Grooved Steel Composite Tube Columns under Axial Compression

**DOI:** 10.3390/polym13213638

**Published:** 2021-10-22

**Authors:** Kunting Miao, Yang Wei, Xi Zhang, Kaiqi Zheng, Fenghui Dong

**Affiliations:** College of Civil Engineering, Nanjing Forestry University, Nanjing 210037, China; njfu_mkt@163.com (K.M.); zx_nfu@163.com (X.Z.); k.zheng@njfu.edu.cn (K.Z.); nldfh@njfu.edu.cn (F.D.)

**Keywords:** FRP, stress-released steel tube, composite structure, axial compression behavior, stress-strain model

## Abstract

A new structure termed “concrete-filled FRP-grooved steel composite tube (CFGCT) column” is proposed, which is composed of a stress-released steel tube (i.e., grooved steel tube), fiber-reinforced polymer (FRP) and concrete. Axial load tests were carried out on twenty-four specimens to investigate the constraint effect of this structure. Three main experimental variables were considered: the steel tube thickness, the FRP type, and the FRP layer. The failure modes, stress-strain relationships and the effect of the main experimental variables were discussed. The stress-strain curves of this new structure are composed of an initial linear part, a nonlinear transition part, a strengthening part and a residual part. The test results demonstrate that the bearing capacity of the structure was improved, and that the mechanical mechanism of the structure was simplified due to the stress-released grooves. Based on the test results and previous studies, formulas for calculating the ultimate stress (*f_cu_*), ultimate strain (*ε_cu_*), peak stress (*f_cc_*) and peak strain (*ε_cc_*) were proposed. In addition, models for predicting the stress-strain curves of CFGCT columns were put forward, and the models could precisely simulate the stress-strain curve of this new composite structure. Hence, this study indicates that a structure composed of FRP and stress-released steel tube can effectively constrain concrete.

## 1. Introduction

Many existing studies show that composite structures can effectively enhance the overall mechanical properties of a structure [1,2,3,4,5]. Currently, composite structures formed by the combination of steel tubes, steel spirals, fiber-reinforced polymer (FRP) and concrete are diffusely applied in civil engineering [6,7,8]. These composite structures can not only be used in the reinforcement of existing structures but can also be applied in new structures. According to relevant studies [9,10], concrete-filled steel tube (CFT) columns can enhance the brittleness and load-bearing capability of concrete structures. However, the durability of a CFT column is still poor, and it is easily subject to local buckling. Furthermore, FRP is diffusely used in civil engineering materials because of its low weight, high strength, great durability and convenience. Therefore, concrete-filled FRP-steel composite tube (CFCT) columns are widely utilized in civil engineering because they combine the common advantages of these two structures [11,12,13,14,15,16,17,18,19,20,21]. Li et al. [22] proposed that the mechanical properties of CFT columns increased because of the constraint provided by the FRP. However, the lateral restraint of the steel tube of most existing CFCT columns does not fully contribute due to the axial yield, which causes a limited increase in the carrying capacity of the specimens. Meanwhile, the stress-strain relationship and constraint efficiency are significant in the study of this kind of structure. The steel tube in a CFCT column is subjected to both a circumferential force and a vertical force; as a result, the stress in the steel tube of a CFCT is complex, and the confinement effect provided by the steel tube on the core concrete is reduced.

To solve these problems, a special type of structure called a steel-tubed concrete column was proposed by Tomii et al. [23] to release the longitudinal stress. Different from ordinary CFT columns, two grooves were cut at both ends of the steel tube. Some experimental investigations have been performed on steel-tubed concrete columns. Wang et al. [24] studied the behavior of slender circular tubed reinforced concrete columns under eccentric load and found that a discontinuous steel tube can improve the ductility of the structure. Qi et al. [25] researched the behavior of tubed steel reinforced-concrete (SRC) stub columns under axial compression. It was concluded that a discontinuous structure can effectively enhance the ultimate strength of the structure. Liu et al. [26] conducted axial compression and eccentric compression tests on 12 tubed SRC columns and found that the bearing capacity of tubed SRC columns is higher than that of ordinary SRC columns. Fiber-based numerical models for this kind of column were also established. Similar conclusions can be found in the studies of Wang et al. [24,26]. Hence, the above researchers have confirmed that discontinuous steel tubes can effectively improve the ductility and bearing capacity of SRC columns. Yang et al. [27] conducted eccentric compression tests on circular CFRP-steel composite tubed SRC columns. The test results indicated that specimens with disconnected CFRP-steel composite tubes showed better ductility and strength. In addition, the deformability also improved with the increase in the number of CFRP layers. Liu et al. [28] studied the axial behavior of circular steel tubed concrete columns confined by a CFRP and found that the grooves at both ends of the steel tube contributed greatly to the bearing capacity and ductility of the specimen. Different models for the ultimate strength of this structure were compared and evaluated. Liu et al. [29] conducted axial compression tests on CFRP-steel composite tubed high-strength concrete columns. Experimental results indicated that the confinement effect of steel tubes can be better utilized in CFRP-steel composite tubed high-strength concrete columns.

**Figure 1:**
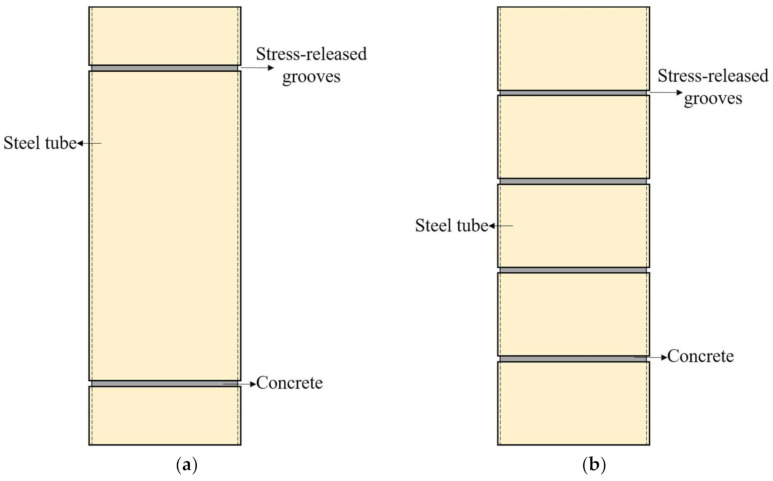
Different kinds of concepts of concrete-filled stress-released steel tubes. (**a**) Steel-tubed concrete columns (**b**) Concrete-filled grooved steel composite tube.

In the above studies, to reduce the influence of an axial load on the steel tube, a groove was slotted at both ends of the specimen (Figure 1a). A better constraint effect has been verified using the above optimized structural details compared to ordinary CFT columns. However, due to the influence of friction, most of the middle part of the height of a steel tubed concrete column is still subjected to longitudinal stress, which makes the constraint effect not ideal, and the internal stress of the steel tube is still complex. To address this gap, more hoop grooves are arranged along the height of the steel tube (Figure 1b) to further reduce or even eliminate the longitudinal stress for the steel tube of the column. Moreover, the mechanical analysis of the structure is also simplified because the steel tube only operates as a hoop constraint.

## 2. New Concrete-Filled FRP-Grooved Steel Composite Tube Columns

**Figure 2:**
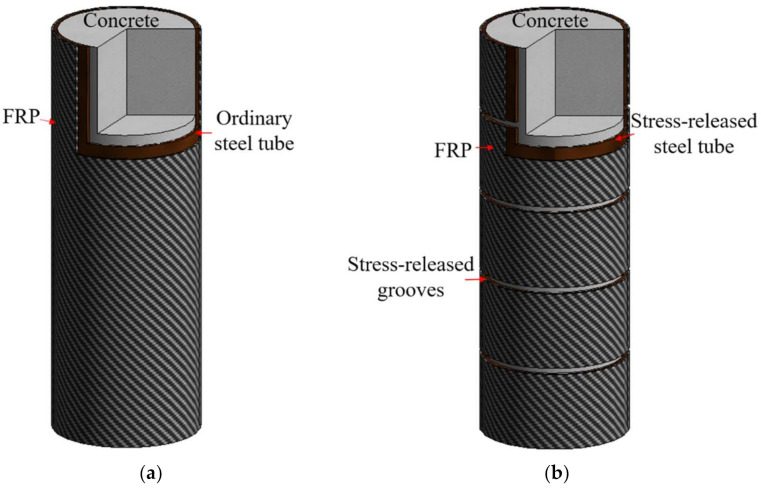
Diagram of two different specimens. (**a**) CFT columns (**b**) CFGCT columns.

Ordinary CFCT columns, as illustrated in Figure 2a, can significantly enhance the ultimate strength and ductility of concrete [30,31,32,33,34]. This structure can be widely applied in high-rise construction structures. However, when this structure is under pressure, the longitudinal buckling of the steel tube usually leads to the failure of the specimen because the steel tube under axial compression more directly bears the vertical compressive stress, in addition to bearing the hoop-tensile stress. Meanwhile, the axial strength of the steel tube is much less than the tensile strength; therefore, when longitudinal buckling of the steel tube occurs, the cyclic direction of the steel tube has not yet reached the yield strength. Therefore, the restraint effect of the steel tube does not fully contribute in ordinary CFCT columns. In addition, when analyzing the stress-strain curve and the confinement effect of ordinary CFCT columns, the steel tube has complex mechanical characteristics as it bears both the circumferential force and the vertical force, so it is difficult to specify the confinement action of the steel tube on the in-filled concrete.

Aiming to address the above problems, a new concrete-filled FRP-grooved steel composite tube (CFGCT) column is proposed (Figure 2b), in which a steel tube is divided into several segments by hoop grooves. The interval grooves release the longitudinal stress of the steel tube when axial loading is applied, ensuring that the steel tube only supplies circumferential confinement for the core concrete during load carrying. A FRP is wrapped on the outside of the steel tube to provide extra strength, so that double confinement can be provided for core concrete. When making CFGCT columns, stress-released grooves are made by assembling steel tube segments and reserving distributed gaps. These steel tube segments are welded to angle steel and fixed temporarily, and polyurethane foam is used to fill the grooves before casting the concrete. Compared with ordinary CFCT columns, the stress-released steel tube does not bear longitudinal pressure, but only bears circumferential tension when the columns are under axial compression. Hence, the longitudinal buckling of the steel tube can be efficiently prevented [32,35]. The stress-released steel tube can definitely offer circumferential confinement for the core concrete, and the force mechanism is clear. Moreover, the effects on the improvement of the concrete strength by the stress-released steel tube are expected to be higher than those of ordinary CFCT columns.

## 3. Experimental Process

### 3.1. Design of the Test

A total of twenty-four CFGCT and ordinary CFT specimens were manufactured in this test. Three ordinary CFT columns were made as contrast specimens. Three concrete-filled grooved steel tube (CFGT) columns and eighteen CFGCT columns were prepared. Three main experimental variables were considered: the steel tube thickness, the FRP type, and the FRP layer. Detailed information on all specimens is shown in Table 1. The labels of the specimens are as follows: the first letter “S” denotes a seamless steel tube, the second number such as “6” denotes the thickness of the steel tube, the third letter denotes the FRP type, where “C” denotes the CFRP-confined specimens, and “B” denotes the BFRP-confined specimens, the latter number denotes the number of FRP layers, the former “N” denotes that the steel tube surface is not wrapped with FRP, and the last “N” means that there is no groove between adjacent steel tubes, and it is an ordinary concrete filled steel tube. For example, “S6C3” represents a specimen of concrete confined with a 6 mm steel tube with grooves and a three-layer CFRP wrapping.

### 3.2. Preparation of Specimens

**Figure 3:**
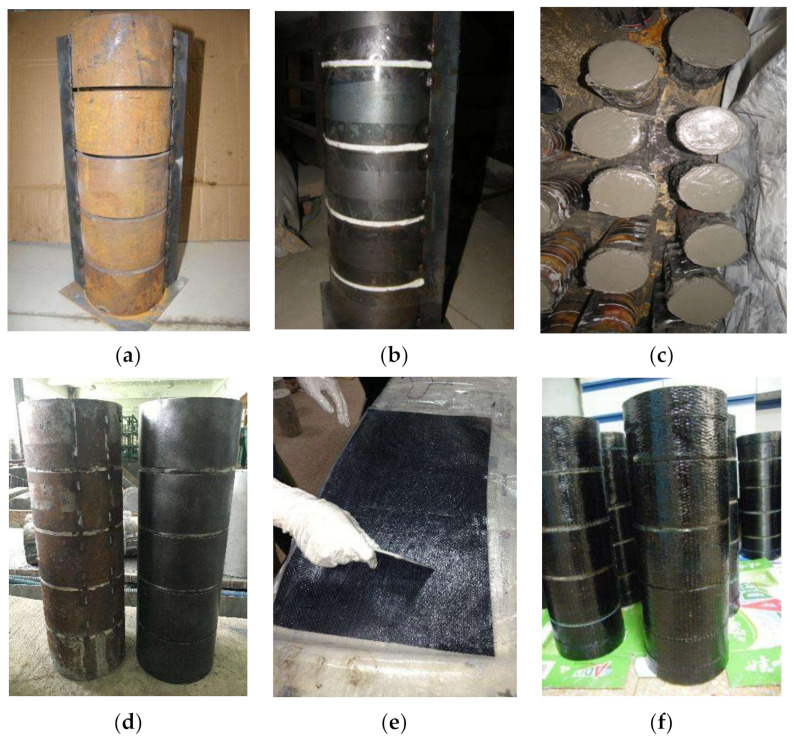
Process of forming the CFGCT columns. (**a**) Setting up the grooved steel tubes. (**b**) Filling in the circumferential gaps. (**c**) Pouring the concrete. (**d**) Surface treatment of steel tubes. (**e**) Impregnating the FRP sheets. (**f**) Wrapping the FRP sheets.

The manufacturing process of the CFGCT column is illustrated in Figure 3. Four main steps were applied to manufacture the CFGCT columns. The first step was the preparation of a stress-released steel tube. First, five steel tubes were placed on the angle iron and fixed by welding. The angle iron was used to ensure a distance of 5 mm between each two steel tubes (as shown in Figure 3a). Then the stress-released grooves were filled by polyurethane foam and sealed with tape to prevent concrete from escaping through the grooves (as shown in Figure 3b). The second step was pouring concrete. Concrete is poured in 2 times and vibrated with a vibrator. The excess concrete in the stress-released grooves was scraped. The specimens were placed in a standard environment for curing for 28 days (as shown in Figure 3c). The third step was surface treatment of the steel tubes. The temporary weld and angle iron were removed by grinding. The surface of the steel tubes was also polished (as shown in Figure 3d). The fourth step was wrapping FRP sheets. The FRP sheets were cut to appropriate size first. The FRP is then impregnated with epoxy resin glue repeatedly. Then the FRP sheet was attached to the steel tube in a circular direction. Finally, the specimen was extruded along the FRP wrapping direction repeatedly to extrude the bubbles (as shown in Figure 3e,f).

### 3.3. Materials

The material properties of concrete are determined by compression tests on three plain concrete columns. The water/cement ratio of concrete was 0.46. The mix ratio of concrete was cement: water: sand: aggregate = 1: 0.46: 1.49: 3.02. The cement used was P. O 42.5. Aggregate used in concrete was gravel with a particle size of 5–10 mm. The average peak stress (*f_co_*) and peak strain (*ε_co_*) were 37.8 MPa and 0.0020, respectively, by testing unconfined concrete cylinders (150 mm × 300 mm) under axial compression. Seamless steel tubes were used in the test. A tensile test was carried out on three standard coupons to obtain the material properties of the steel tubes. The average yield strengths of the different steel tubes were 283.9 MPa, 304.8 MPa and 365.0 MPa, respectively. The average elastic modulus of the different steel tubes was 2.00 GPa, 1.90 GPa and 1.92 GPa, respectively. The mechanical properties of both types of FRP, as shown in Table 2, were determined by five coupon tests. The epoxy resin (L500-AS/L-500BS) was blended uniformly in a 1:2 ratio for use.

### 3.4. Test Setup and Instruments

**Figure 4:**
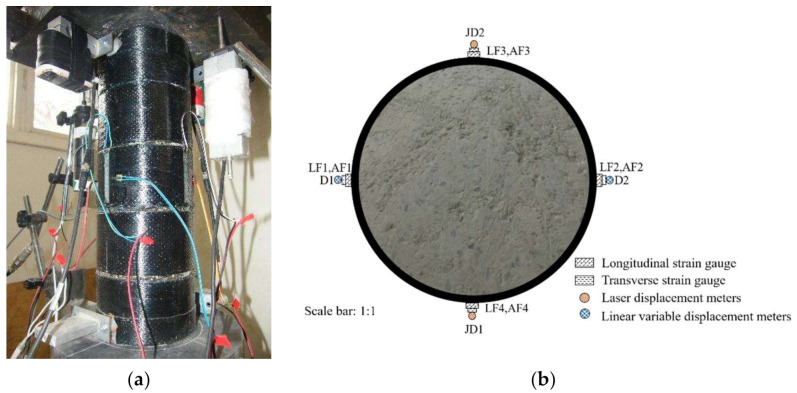
Test arrangement and instruments. (**a**) Test setup. (**b**) Layout of instruments.

A 300 t testing machine shown in Figure 4a was used in the test. The layouts of the linear variable displacement meters (D1 and D2), laser displacement meters (JD1 and JD2) and strain gauges are shown in Figure 4b. Before testing, 20% of the ultimate load was preloaded to ensure that the specimen and the testing machine were in close contact, and the equipment was adjusted. The initial rate was 0.5 mm/min. After the FRP was broken, the rate increased to 0.8 mm/min. The test data (axial load, deformation and strain) were collected using a TDS-530 data logger.

## 4. Experimental Phenomena and Data Analysis

### 4.1. Failure Modes

#### 4.1.1. CFT Columns

**Figure A1:**
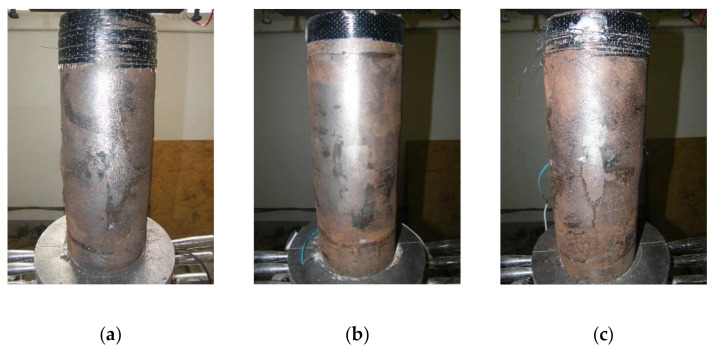
Failure modes of the CFT columns. (**a**) S4.5NN (**b**) S6NN (**c**) S7.5NN.

For the CFT columns (i.e., Specimens S4.5NN, S6NN and S7.5NN), as shown in Figure A1 in Appendix A, at the beginning, the surface of the CFT columns showed no clear change. As the load increased, the FRP on the end of the specimens broke slightly, and the steel tube in the middle part of the specimen buckled at first. Then, the buckling began to spread to the upper part or lower part of the specimen as the loading increased. At the later loading stage the middle lower part and the middle upper part of the specimens both showed buckling. Moreover, the failure modes of specimens with thicker steel tubes were less serious.

#### 4.1.2. CFGT Columns

**Figure A2:**
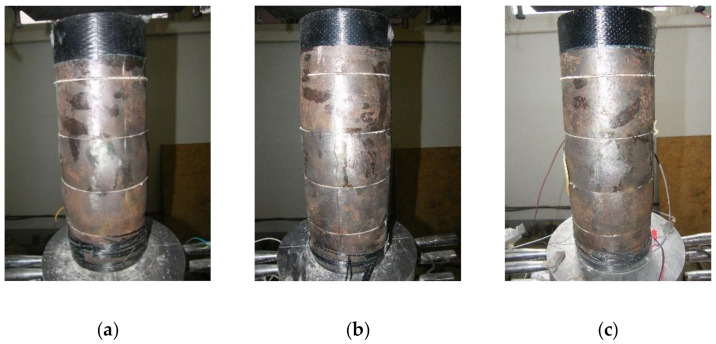
Failure modes of the CFGT columns. (**a**) S4.5N (**b**) S6N (**c**) S7.5N.

Similar to the ordinary CFT columns, the surface of the CFGT columns (i.e., Specimens S4.5N, S6N and S7.5N) did not change significantly at the beginning, as shown in Figure A2 in Appendix A. With increasing load, the width of the stress-released groove decreased. A small amount of concrete also fell off from the stress-released groove. When the load approached the peak point, the stress-released grooves almost disappeared. Local bulges also appeared in the middle of the steel tube afterward. At the later stage of loading, the steel tube seriously buckled, and the deformation of the steel tube on both sides of the stress-released grooves was the most serious.

#### 4.1.3. CFGCT Columns

**Figure A3:**
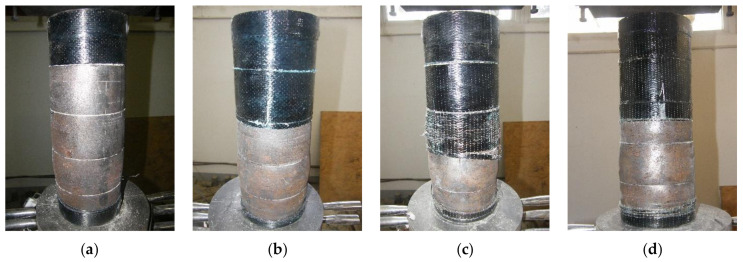

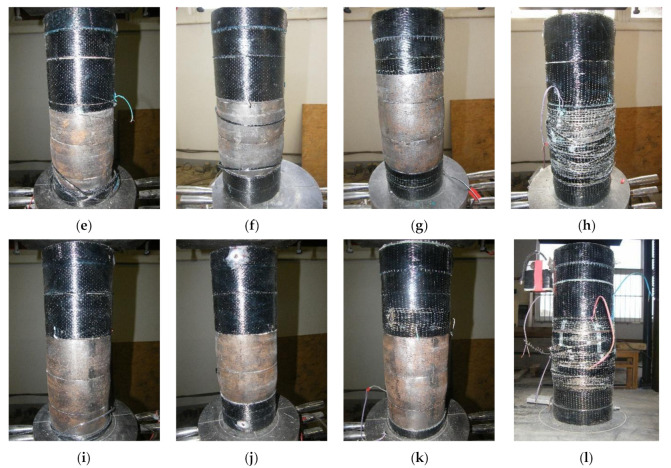
Failure modes of the CFGCT columns. (**a**) S4.5C1. (**b**) S4.5C3. (**c**) S4.5B2. (**d**) S4.5B3. (**e**) S6.0C2. (**f**) S6.0C3. (**g**) S6.0B1. (**h**) S6.0B3. (**i**) S7.5C1. (**j**) S7.5C2. (**k**) S7.5B1. (**l**) S7.5B3.

The failure of the CFGCT columns was always caused by the breakage of the FRP sheets, as shown in Figure A3 in Appendix A. At the beginning of the test, the surface of the CFGCT columns (i.e., Specimens S4.5B, S4.5C, S6B, S6C, S7.5B, and S7.5C) did not show significant changes. With increasing load, the stress-released grooves became narrower. A small amount of concrete also fell off from the stress-released grooves. When the load reached 80%-90% of the ultimate load, the stress-released grooves clearly disappeared. The FRP in the middle of the specimen began to rupture and break away due to the buckling of the steel tube. When the FRP in the middle of the specimen completely fell off, the FRP in the upper part or the lower part of the specimen also slightly fell off, indicating that the transverse deformation of the concrete in the middle of the specimen was large during the compression process. Different from the slight failure mode of the BFRP-wrapped specimens, the failure of the CFRP-wrapped specimens was more severe, showing filiform fracture, while the BFRP flaked off.

### 4.2. Stress-Strain Relationship

**Figure 5:**
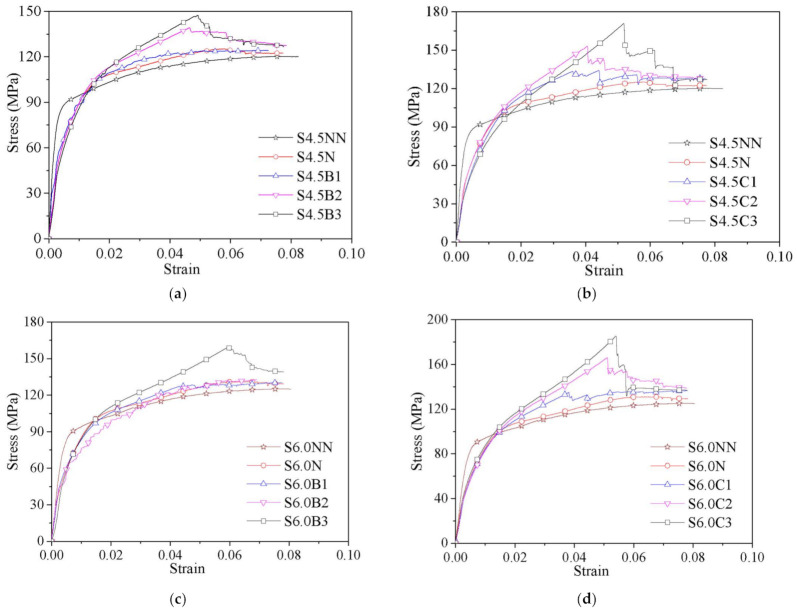

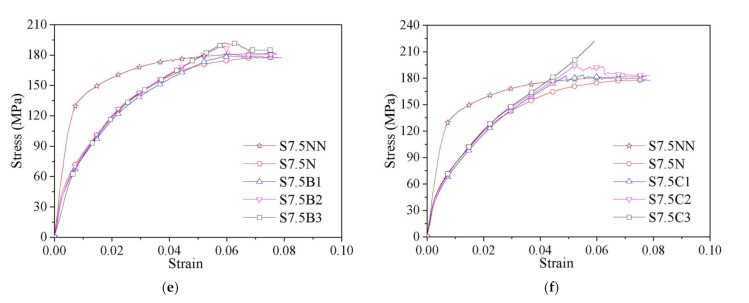
Stress-overall strain curves of the specimens. (**a**) S4.5B series. (**b**) S4.5C series. (**c**) S6B series. (**d**) S6C series. (**e**) S7.5B series. (**f**) S7.5C series.

**Figure 6:**
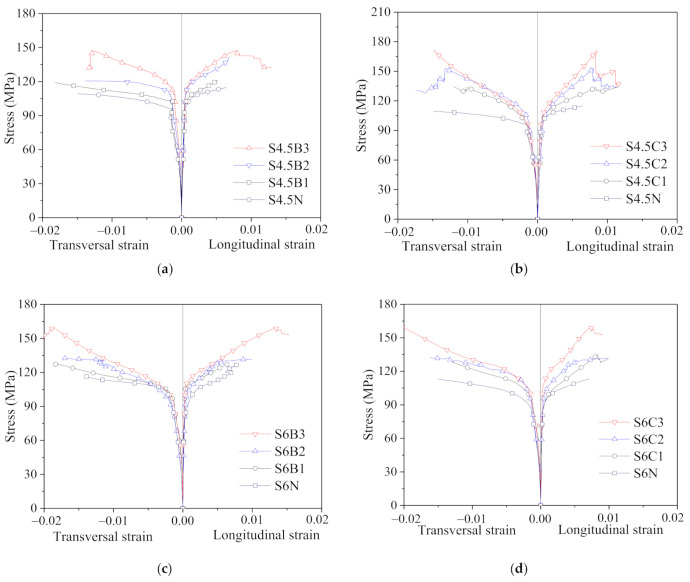

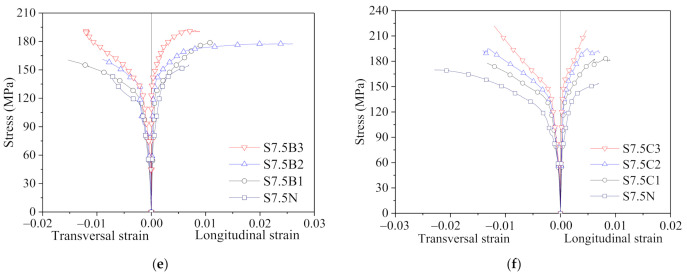
Stress-local strain curves of specimens. (**a**) S4.5B series. (**b**) S4.5C series. (**c**) S6B series. (**d**) S6C series. (**e**) S7.5B series. (**f**) S7.5C series.

The strain measured by the linear variable displacement meters or laser displacement meters was much greater than that measured by the longitudinal strain gauge due to the existence of the stress-released grooves. Therefore, both the stress-overall strain curve (Figure 5) and the stress-local strain curve (Figure 6) of the specimens were recorded and compared. The stress of both curves is equal to the axial bearing capacity divided by the cross-sectional area of the specimen. The overall strain is calculated by using the displacement measured by the linear variable displacement meters and laser displacement meters divided by the measurement range, while the local strain is measured by a longitudinal strain gauge pasted on the center region of the steel tube.

**Figure 7:**
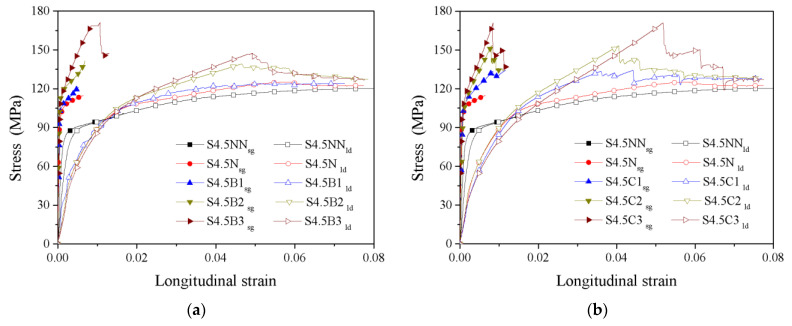

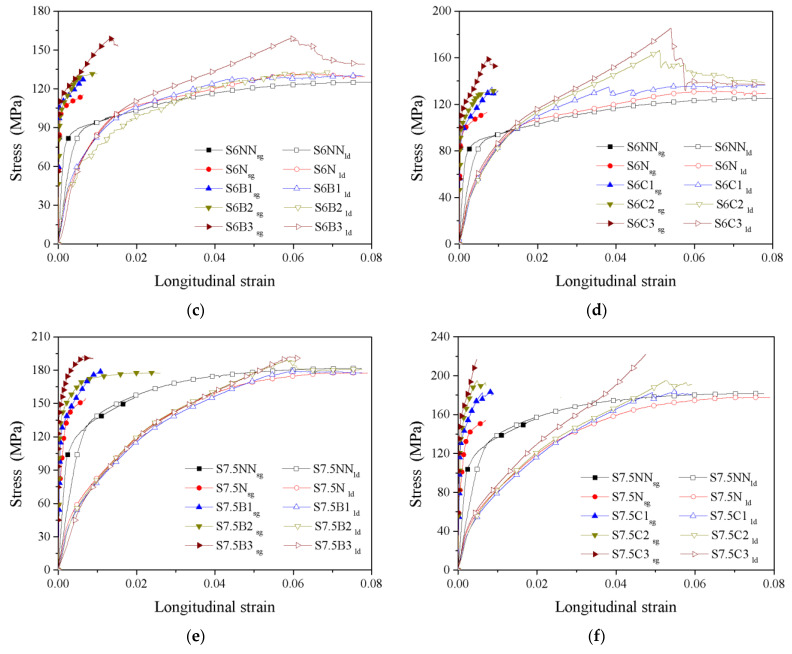
Comparison of the stress-overall strain and stress-local strain curves. (**a**) S4.5B series. (**b**) S4.5C series. (**c**) S6B series. (**d**) S6C series. (**e**) S7.5B series. (**f**) S7.5C series.

The stress-overall strain and stress-local strain curves of all specimens are compared in Figure 7. Clearly, the stress-overall strain and stress-local strain curves of the CFGCT columns show a significant difference because the steel tube is composed of discrete sections and does not bear the axial stress in the initial stage due to the existence of stress-released grooves. The deformation measured by the strain gauge will be much smaller than that measured by displacement. Hence, the slope of the initial stage in the stress-strain curves measured by the strain gauge will be much larger than that of the stress-strain curves obtained by measuring the displacement. Moreover, the ultimate strain and peak strain measured by the longitudinal strain gauge or the displacement meters are different, as shown in Table 3.

#### 4.2.1. Stress-Overall Strain Curves

The stress-overall strain curves of all specimens are compared in Figure 5. The stress-overall strain curves of CFT columns (i.e., Specimens S4.5NN, S6NN and S7.5NN) and the CFGT columns (i.e., S4.5N, S6N and S7.5N) exhibited an initial linear part, elastic-plastic part and residual part. In the initial linear part, the stress increased linearly with strain. However, the initial stiffness of the CFT columns was larger than that of the CFGT columns with the same steel tube thickness. This is because for the CFGT columns, only the concrete bears the axial load in the initial stage due to the stress-released grooves, and the steel tube begins to bear the axial load after the stress-released grooves are gone. In the elastic-plastic part, the rate of the load increase began to decrease, and local bulges began to appear in the middle of the steel tube. After the specimens reached the ultimate state, the stress-overall strain entered the residual part. In this part, the bulges of the steel tube became more serious, but the stress remained almost unchanged.

The stress-overall strain curves of the CFGCT columns are composed of an initial linear part, a nonlinear transition part, a strengthening part and a residual part. In the initial linear part, the curves of the CFGT columns and the CFGCT columns are almost identical, indicating that the confinement provided by the FRP is not clear in the initial stage. After a nonlinear transition part where the steel tube yields, a strengthening part with a smaller slope appears. The strengthening part is reflected only in the stress-overall strain curve of the CFGCT columns, which indicates that the FRP still provides a confinement effect after the steel tube yields. Moreover, the slope of the strengthening part was related to the layers of FRP. The more FRP layers there are, the greater the slope of the strengthening part is. It can also be found that the CFGCT columns wrapped with CFRP show significant difference in the slopes of the nonlinear transition part and the strengthening part with the increasing number of FRP layers, which is not clear in the CFGCT columns wrapped with BFRP. This phenomenon can be attributed to the fact that CFRP has a higher elastic modulus than BFRP. Finally, when the specimens reach the limit state, the stress drops to a certain value and enters the residual part. In the residual part, the specimens with more FRP layers showed better residual strength.

#### 4.2.2. Stress-Local Strain Curves

Figure 6 shows the stress-local curves of the CFGT columns. The longitudinal strain of the curve is measured by a longitudinal strain gauge, and the transverse strain of the curve is measured by a transverse strain gauge. The stress-longitudinal strain curves of the CFGCT columns are composed of four parts: the initial part, the nonlinear transition part, the strengthening part and the residual part. In the initial part, the curves of the CFGCT specimens were almost the same as those of the plain concrete specimens. With increasing load, the curves entered the nonlinear transition part and presented as nonlinear elastoplastic. In the strengthening part, the curves displayed a straight line, and the slope of the curves in this stage increased with the increase in the number of FRP layers. In the residual part, the core concrete was only confined by steel tubes after the FRP fractured.

The stress-transverse strain curves of the CFGCT columns can be divided into three stages. In the first stage, similar to the stress-longitudinal strain curves, the stress-transverse strain curves of the specimens with the same steel tube thickness are almost identical. With increasing load, the stress-transverse strain of the specimens with different layers began to show differences. In the case of the load being the same, the transverse strain of the specimens with more FRP layers was small, reflecting that the confinement effect provided by the FRP can moderate the transverse deformation of the concrete. After the load reached the peak point, the stress-transverse strain curves entered the linear strengthening stage. In this stage, the slope of the stress-transverse strain curves of the specimens with more FRP layers was larger, while the growth rate of the transverse deformation of the specimens with more FRP layers was slower, indicating that the confinement effect provided by the FRP of the specimens with more FRP layers was more significant.

### 4.3. Parameter Analysis

#### 4.3.1. Effect of the Stress-Released Grooves

**Figure 8:**
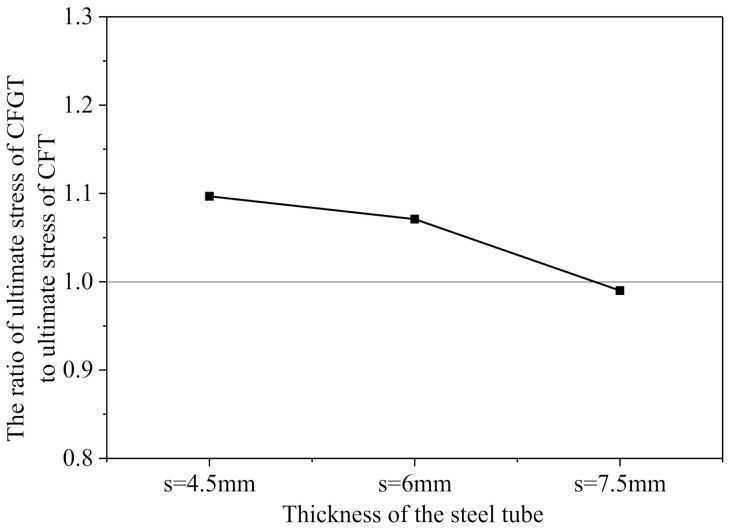
Strength ratio of concrete confined with steel tubes with grooves and without grooves.

The effect of the stress-released grooves on the mechanical properties of the structures is mainly reflected in the initial stiffness and the ultimate strength of the specimen. As seen in Figure 8, the initial stiffness of the ordinary CFT specimens is clearly larger than that of the CFGT specimens. This can be caused by the fact that the steel tubes in the ordinary CFT columns bear the vertical loads directly, while the steel tubes in the CFGT columns did not bear the vertical load until the stress-released grooves disappeared.

Figure 8 shows the comparison of the ratio of the strength of the CFGT columns to that of the CFGT columns. When the thickness of the steel tube was thin, the ultimate strength of the CFGT columns (S4.5N and S6.0N) was greater than that of the CFT columns (S4.5NN and S6.0NN). The ultimate bearing capacity of S4.5N was 1.09 times that of S4.5NN, and the ultimate bearing capacity of S6N was 1.07 times that of S6NN. When the steel tube was thicker, the ultimate strength of the CFT columns (S7.5NN) was almost equal to that of the CFGT columns (S7.5N). The ultimate bearing capacity of S7.5N was 0.99 times that of S7.5NN. Hence, it can be concluded that the stress-released grooves can only play a positive role in specimens with smaller steel tube thicknesses.

#### 4.3.2. Effect of the FRP Layers

As shown in Figure 6, in the initial part, the curves of specimens with different FRP layers are almost the same. In the strengthening part, the curves of various specimens were different. The higher the number of FRP layers, the larger the slope of the strengthening in the stress-overall strain curve. Moreover, the strengthening effect provided by the increase in the number of FRP layers is more significant for the specimens with thinner steel tubes. Finally, the curve entered the residual stage after the FRP broke, and the core concrete reverted to the stage where it was only restrained by the steel tube. Therefore, in the residual stage, the change in the number of FRP layers barely affects the mechanical properties.

**Figure 9:**
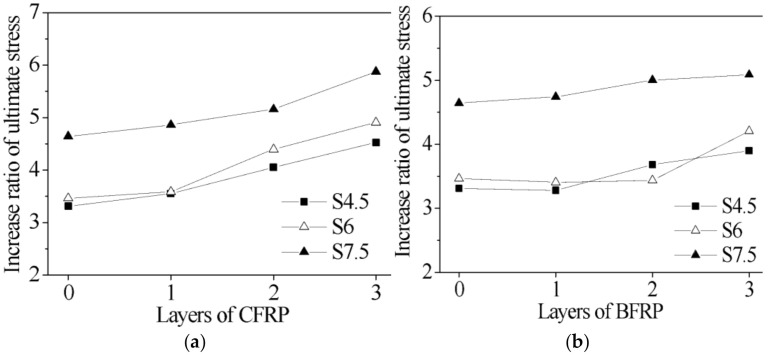
Effect of the number of layers of FRP on the ultimate stress. (**a**) Specimens wrapped with CFRP. (**b**) T Specimens wrapped with BFRP.

The increased ratio of the ultimate stress of all specimens is demonstrated in Figure 9. The increase in the number of layers of FRP can significantly increase the ultimate stress. From Figure 9a, for the CFGCT columns (CFRP-wrapped) with a 4.5 mm steel tube thickness, when the number of FRP layers increased from 0 to 3, the ultimate stress of the specimens improved by 7.26%, 14.16% and 11.60%, respectively. For the CFGCT columns (CFRP-wrapped) with a 6.0 mm steel tube thickness, when the number of FRP layers increased from 0 to 3, the ultimate stress of the specimens improved by 3.49%, 22.66% and 11.58%, respectively. For the CFGCT columns (CFRP-wrapped) with a 7.5 mm steel tube thickness, when the number of FRP layers increased from 0 to 3, the ultimate stress of the specimens improved by 4.73%, 6.10% and 13.89%, respectively. From Figure 9b, when the number of FRP layers increased from 1 to 3, the ultimate stress of the CFGCT columns (BFRP-wrapped) with steel tube thicknesses of 4.5 mm, 6.0 mm and 7.5 mm increased from 5.91% to 12.26%, 0.92% to 22.41% and 1.74% to 5.49%, respectively. Hence, it can be concluded that the increase in the number of FRP layers can significantly improve the stiffness and ultimate stress of the specimens.

#### 4.3.3. Effect of the FRP Type

**Figure 10:**
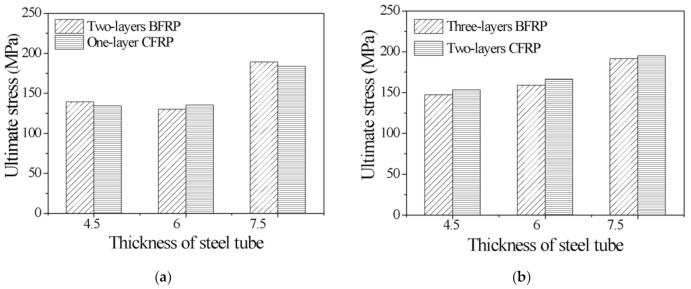
Effect of the FRP type on the ultimate stress. (**a**) Two-layers BFRP and one-layer CFRP. (**b**) Three-layers BFRP and two-layers CFRP.

**Figure 11:**
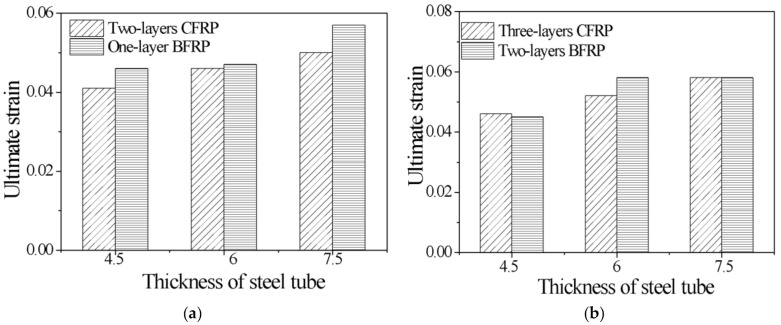
Effect of the FRP type on the ultimate strain. (**a**) Two-layers BFRP and one-layer CFRP. (**b**) Three-layers BFRP and two-layers CFRP.

According to the material test, the ultimate strength of the CFRP was greater than that of the BFRP, and the ultimate strain of the BFRP was greater than that of the CFRP. Therefore, in Figure 10 and Figure 11, the ultimate stress and ultimate strain of specimens with different FRP types are compared.

Figure 10 compares the ultimate stress of specimens with different FRP types. The ultimate stresses of S4.5B2 and S4.5C1 are approximately 3.5 times that of the unconfined concrete columns. The ultimate stresses of S6.0B2 and S6.0C1 are 3.5 times that of the unconfined concrete columns. The ultimate stresses of S7.5B2 and S7.5C1 are 5.0 times that of the unconfined concrete columns. Hence, it can be concluded that for the CFGCT columns, the ultimate stresses with one layer of CFRP and two layers of BFRP are almost equal. The same conclusion can be drawn for the specimens with two layers of CFRP and three layers of BFRP. The ultimate stresses of S4.5B3 and S4.5C2 are 147.42 MPa and 153.21 MPa, respectively. The ultimate stresses of S6.0B3 and S6.0C2 are 158.99 MPa and 166.41 MPa, respectively. The ultimate stresses of S7.5B3 and S7.5C2 are 192.28 MPa and 195.00 MPa, respectively. Therefore, in the case where the other parameters are all the same, the CFGCT columns (CFRP-wrapped) show better bearing capacity due to the higher elastic modulus of the CFRP.

Figure 11 shows the difference between the ultimate strain of specimens with different FRP types. As seen in Figure 11, for the CFGCT columns, the ultimate strain of specimens with one layer of BFRP is greater than that of specimens with two layers of CFRP, and the ultimate strain of the specimens with two layers of BFRP is basically the same as that of the specimens with three layers of CFRP. For example, the ultimate overall strains of S4.5B1 and S4.5C2 are 0.046 and 0.041, respectively. The ultimate overall strains of S4.5B2 and S4.5C3 are 0.045 and 0.046, respectively. Therefore, in the case where the other parameters are all the same, the CFGCT columns (BFRP-wrapped) show better deformation capacity due to the better ductility of the BFRP.

## 5. Analytical Modeling

### 5.1. Stress-Strain Model

#### 5.1.1. Stress-Strain Relationship Curves

**Figure 12:**
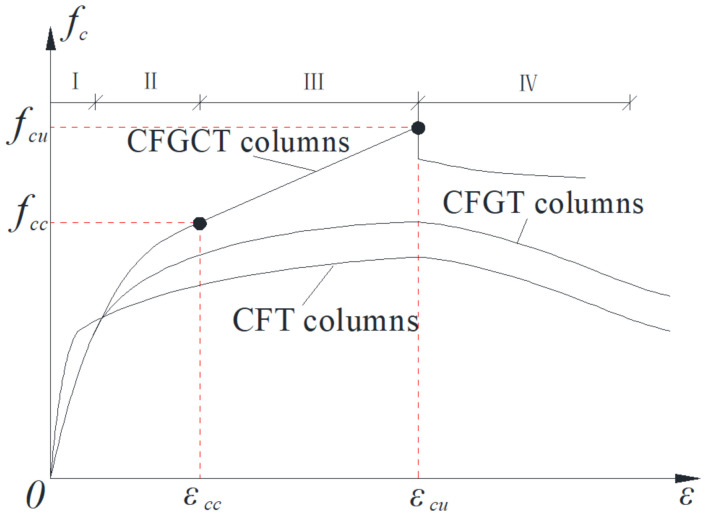
Stress-strain curve models of confined concrete.

The typical stress-strain curves of concrete-filled steel tube (CFT) columns, concrete-filled grooved steel composite tube (CFGT) columns and CFGCT columns are summarized in Figure 12. In general, the stress-strain curve of the CFT and CFGT columns is composed of the initial part, the elastic-plastic part and the residual stage, and the stress-strain curve of the CFGCT columns is composed of the initial linear part, the nonlinear transition part, the strengthening part and the descending part. In the initial part, the curves of the plain concrete, CFGT and CFGCT columns are almost the same. However, the initial stiffness (i.e., the slope of the initial part) of the CFT columns is larger than that of the CFGT and CFGCT columns because the steel tubes of the CFGT and CFGCT columns do not bear the load in the initial stage due to the stress-released grooves. With increasing axial load, the curves of the CFT, CFGT and CFGCT columns show different trends because of the different constraints on the concrete. For the CFT and CFGT columns, after reaching the peak point, the steel tube yields, and the upward trend of the curve slows down. However, the steel tube can still provide a constant circumferential constraint after yielding, and the specimen can maintain a certain residual bearing capacity and show good ductility. For the CFGCT columns, after the steel tube yields, the curves enter a second linear strengthening stage due to the transverse restraint provided by the FRP. When the load reaches the ultimate load, the FRP breaks and the core concrete is constrained by the steel tube only, and the curves enter the residual stage.

#### 5.1.2. Calculating the Model

Based on the general stress-strain model of the CFT proposed by Wu and Wei [36], some improvements are applied to this model by analyzing the test results:

(1) $$f(x)=\frac{f_{c}}{f_{cc}}=\frac{(x)\cdot a}{a-1+{\left(x\right)}^{a{\left(x+\delta \right)}^{b}+c}}$$

where *x*= *ε_c_*/*ε_cc_* (*ε_cc_* is the peak strain); *a* can be calculated by Equation (2), in which *E_cs0_* represents the initial elastic modulus and can be calculated by Equation (3); *b* and *c* are two additional coefficients to make the formula more universal; *b* is set to a constant value of −0.1; *δ* is set to a small constant value of 0.01; and *c* can be calculated by the formula in the Wu and Wei [36] model.

(2) $$a=\frac{E_{cs0}}{E_{cs0}-\frac{f_{cc}}{\varepsilon_{cc}}}$$

(3) $$E_{cs0}=\mu_{1}(\frac{A_{c}E_{c}+\mu_{2}A_{s}E_{s}}{A_{g}})$$

where *E_c_* is the elasticity modulus of concrete and *E_s_* is the elasticity modulus of steel.

*μ_1_* and *μ_2_* are 4.45 and 1.45 and 0.47 and 0.30 for the stress-local strain curve and the stress-overall strain curve, respectively, obtained by regression analysis.

### 5.2. Key Parameters of the Model

The steel tube of this structure does not bear axial load directly due to its discontinuity and only plays a role as a hoop constraint; hence, the confinement effect of the stress-released steel tube on the core concrete is similar to that of a steel spiral. Therefore, the confinement efficiency of the CFGCT columns can be calculated using the model of concrete-filled FRP-steel spiral composite tube columns. This test summarizes the data of other researchers to build a database, as shown in Table 4. The main parameters considered in the test are the concrete strength (27.6–55.0 MPa), diameter (150–303 mm), type of FRP (CFRP, BFRP and GFRP), steel spiral spacing, number of FRP layers, etc. [37,38,39,40,41,42,43]. In addition, three indices were used to evaluate the proposed model: the average value (*AV*), standard deviation (*SD*), and average absolute error (*AAE*).

#### 5.2.1. Peak Stress and Peak Strain

Equations (4) and (5) were proposed to estimate the *f_cc_* and *ε_cc_* of concrete confined by a FRP [44]:

(4) $$\frac{f_{cc}}{f_{co}}=1+0.0015\frac{E_{l}}{f_{co}{}^{0.5}}$$

(5) $$\frac{\varepsilon_{cc}}{\varepsilon_{co}}=1+0.003\frac{E_{l}}{f_{co}{}^{0.5}}$$

Equations (6) and (7) were suggested to calculate the *f_cc_* and *ε_cc_* of concrete confined by steel stirrups [45]:

(6) $$\frac{f_{cc}}{f_{co}}=1+5.35\frac{f_{ls}{}^{0.86}}{f_{co}}$$

(7) $$\frac{\varepsilon_{cc}}{\varepsilon_{co}}=1+20.6(\frac{f_{ls}}{f_{co}})$$

For the CFGCT columns, the peak stress *f_cc_* can be obtained by summing the confinement effect from the FRP and the stress-released steel tube. However, due to the roles of the concentrated deformation by the grooves, the overall peak strain (*ε_cc,a_*) is clearly smaller than those of common concrete with an equivalent confining condition with an identical amount of steel and FRP. Therefore, the local peak strain of the CFGCT columns can be evaluated by reducing the sum of the confinement effect from the FRP and the stress-released steel tube using a reduced coefficient.

(8) $$\frac{f_{cc}}{f_{co}}=1+0.0015\frac{E_{l}}{f_{co}{}^{0.5}}+5.35\frac{f_{ls}{}^{0.86}}{f_{co}}\frac{A_{c}}{A_{g}}$$

(9) $$\frac{\varepsilon_{cc,l}}{\varepsilon_{co}}=1+0.003\alpha \frac{E_{l}}{f_{co}{}^{0.5}}+20.6\beta \frac{f_{ls}}{f_{co}}\frac{A_{c}}{A_{g}}$$

where

(10) $$f_{ls}=\frac{2t_{se}f_{y}}{D-2t_{se}}$$

(11) $$t_{se}=\frac{L-n_{g}t_{g}}{L}t_{s}$$

(12) $$E_{l}=\frac{2E_{f}t_{f}}{D}$$

where, *f_ls_* is the confinement pressure provided by stress-released steel tube (Equation (11)); *ε_cc,l_* is the local peak stress of the CFGCT columns; *f_y_* is the yield strength of the steel tube; *E_f_* is the modulus of elasticity of the FRP; *n_g_* is the number of grooves; *t_g_* is the width of the grooves; *t_s_* is the thickness of the steel tube; *D* is the diameter of the column; the reduced coefficients *α* and *β* are 0.08 and 0.012, respectively, according to the test results.

Due to the structural details of the stress-released grooves, the local peak strain (*ε_cc,a_*) of the CFGCT columns is clearly smaller than those of common concrete with an equivalent confining condition and an identical amount of steel and FRP, and concentrated longitudinal deformation could occur at the grooves. The experimental result indicated that the local peak strain (*ε_cc,l_*) is linearly related to the overall peak strain (*ε_cc,a_*) for the CFGCT columns; therefore, *ε_cc,l_* can be determined using the following formula:

(13) $$\varepsilon_{cc,a}=5.2\varepsilon_{cc,l}+0.01$$

**Figure 13:**
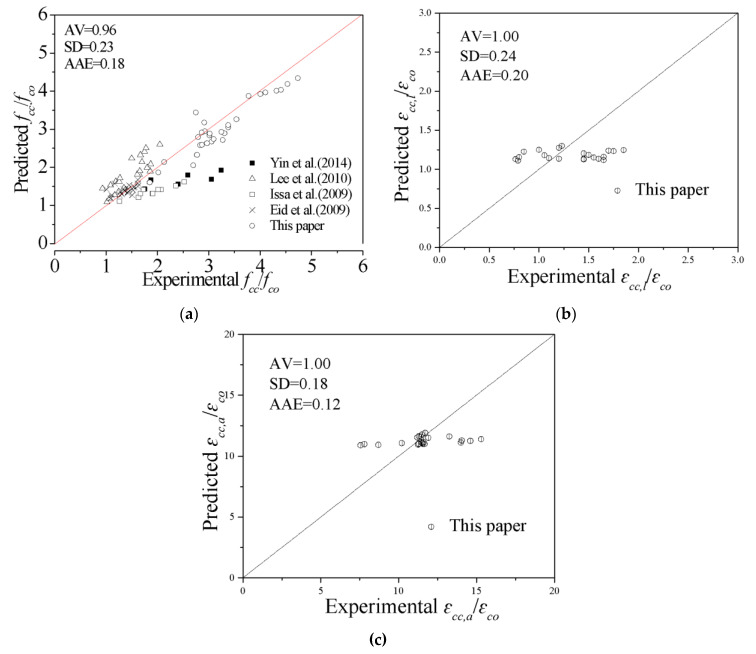
Evaluation of the peak point models. (**a**) Peak stress (**b**) Local peak strain (**c**) Overall peak strain.

Verification of the peak point models is shown in Figure 13. The proposed models can predict the database in this test well. The *AV*, *SD*, and *AAE* of the ratio of the predicted peak stress to the experimental peak stress are 0.96, 0.23 and 0.18, respectively. The *AV*, *SD*, and *AAE* of the ratio of the predicted local peak strain to the experimental local peak strain are 1.00, 0.24 and 0.20, respectively. The *AV*, *SD*, and *AAE* of the ratio of the predicted overall peak strain to the experimental overall peak strain are 1.00, 0.18 and 0.12, respectively.

#### 5.2.2. Ultimate Stress and Ultimate Strain

Equations (14) and (15) were proposed to estimate *f_cu_* and *ε_cu_* of concrete confined by a FRP [44]:

(14) $$\frac{f_{cu}}{f_{co}}=0.75+2.7{\left(\frac{f_{lf}}{f_{co}}\right)}^{0.9}$$

(15) $$\frac{\varepsilon_{cu}}{\varepsilon_{co}}=1.75+140\left(\frac{f_{lf}}{f_{co}}\right)\varepsilon_{fu}{}^{0.6}$$

The following equation was suggested to estimate the *f_cu_* of concrete confined by steel stirrups [36,46]:

(16) $$f_{cu}=f_{co}+4.1f_{ls}$$

Similar to the peak stress and local peak strain, the ultimate stress and local peak strain could be obtained by summing the confinement effect from the FRP and the stress-released steel tube and considering adjustment coefficients for the CFGCT columns. The ultimate stress (*f_cu_*) and local ultimate strain (*ε_cu,l_*) can be determined by the following formulas:

(17) $$\frac{f_{cu}}{f_{co}}=0.75+2.7\gamma {\left(\frac{f_{lf}}{f_{co}}\right)}^{0.9}+4.1\frac{f_{ls}}{f_{co}}\cdot \frac{A_{c}}{A_{g}}$$

(18) $$\frac{\varepsilon_{cu,l}}{\varepsilon_{co}}=1.75+140\lambda_{1}\left(\frac{f_{lf}}{f_{co}}\right){\left(\varepsilon_{fu}\right)}^{0.6}+20.6\lambda_{2}\left(\frac{f_{ls}}{f_{co}}\right)\frac{A_{c}}{A_{g}}$$

where the coefficients *γ*, *λ_1_* and *λ_2_* are 1.03, 0.42 and 0.22, respectively, from regression analysis of the test results; *ε_fu_* is the ultimate strain of the FRP; and *f_lf_* is the confining pressure provided by the FRP, determined through the following equation:

(19) $$f_{lf}=\frac{2E_{f}\varepsilon_{fu}t_{f}}{D}$$

The local ultimate strain (*ε_cu,l_*) is linearly related to the overall ultimate strain (*ε_cu,a_*) for the CFGCT columns and can be given by:

(20) $$\varepsilon_{cu,a}=1.3\varepsilon_{cu,l}+0.04$$

**Figure 14:**
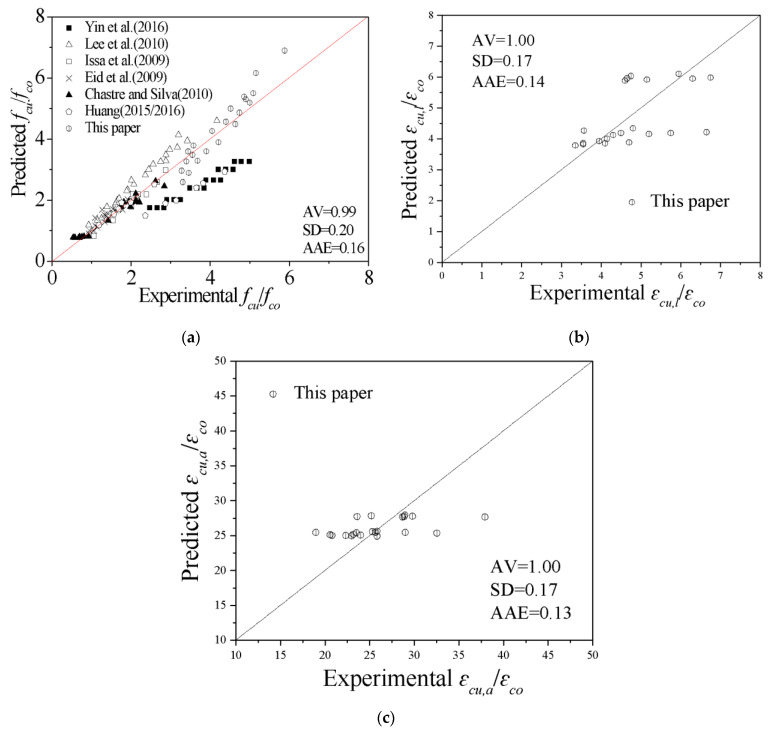
Evaluation of the ultimate point models. (**a**) Ultimate stress. (**b**) Local ultimate strain. (**c**) Overall ultimate strain.

Verification of the ultimate point models is given in Figure 14. The proposed ultimate stress model can predict the database of this paper well, while the test data of Yin et al. and Huang et al. [42,43] are below the ideal line. This issue can be explained by the fact that GFRPs are used as confined material in their tests, indicating that the prediction of the model of GFRP specimens is conservative. The *AV*, *SD*, and *AAE* of the ratio of the predicted ultimate stress to the experimental peak stress are 0.99, 0.20 and 0.16, respectively. The *AV*, *SD*, and *AAE* of the ratio of the predicted local ultimate strain to the experimental local peak strain are 1.00, 0.17 and 0.14, respectively. The *AV*, *SD*, and *AAE* of the ratio of the predicted overall ultimate strain to the experimental overall peak strain are 1.00, 0.17 and 0.13, respectively.

### 5.3. Prediction of the Stress-Strain Curves

**Figure 15:**
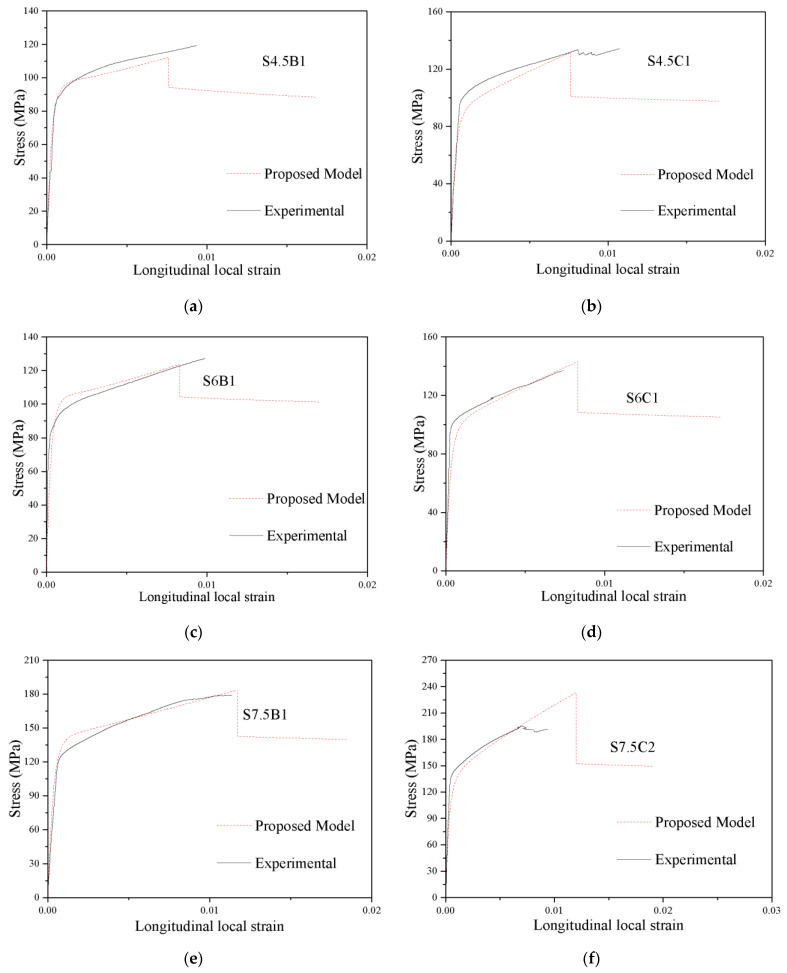
Evaluation of stress-local strain curves of CFGCT columns. (**a**) S4.5B1. (**b**) S4.5C1. (**c**) S6B1. (**d**) S6C1. (**e**) S7.5B1. (**f**) S7.5C2.

**Figure 16:**
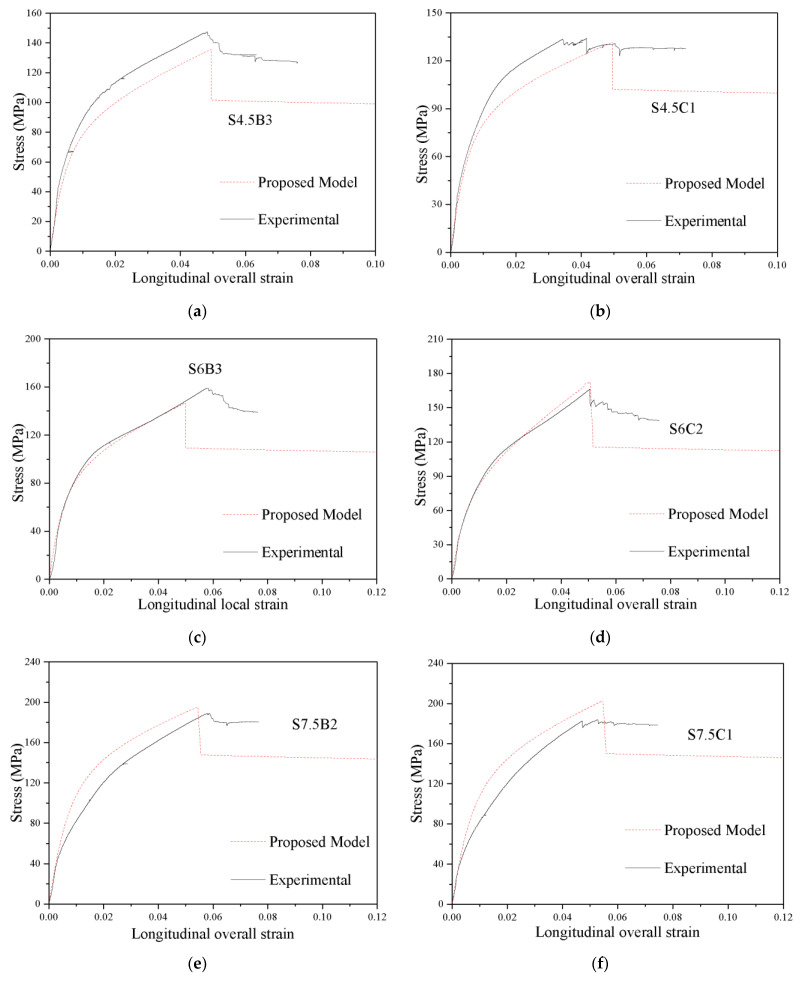
Evaluation of stress-overall strain curves of CFGCT columns. (**a**) S4.5B3. (**b**) S4.5C1. (**c**) S6B3. (**d**) S6C2. (**e**) S7.5B2. (**f**) S7.5C1.

The predicted values of *f_cu_*, *ε_cu_*, *f_cc_* and *ε_cc_* were substituted into Equation (1) to obtain the parameters of the model, and then the theoretical stress-strain curve of each specimen was calculated. Part of the comparison of the predicted stress-strain curve and the experimental stress-strain curve of the specimens in this paper is shown in Figure 15 and Figure 16. From Figure 15 and Figure 16, it can be concluded that the experimental stress-local/overall strain curve and the proposed stress-local/overall strain curve of the CFGCT columns are in good agreement. The initial linear part, nonlinear transition part, strengthening part and descending part can be obtained using the proposed model for CFGCT columns. Hence, the proposed model can simulate the stress-overall strain curve and stress-local strain curve of CFGCT columns well. Moreover, the expression in the proposed model is simple and general, which facilitates excellent flexibility and generality in practical applications.

## 6. Conclusions

A new structure termed concrete-filled FRP-grooved steel composite tube (CFGCT) columns is proposed, and eighteen CFGCT columns, three CFGT columns and three CFT columns are tested under axial compression to investigate the confinement effect. Furthermore, stress-strain models considering the influence of the stress-released grooves were proposed based on the test results. The following conclusions can be drawn:

(1) Compared with the CFT columns, the initial stiffness of the CFGT columns is smaller due to the stress-released grooves. The bearing capacity and deformation of the CFGT columns are significantly improved because of the existence of the stress-released grooves. In addition, the steel tube only plays a role as a hoop constraint and does not bear the longitudinal pressure, so the mechanical mechanism of the structure is simplified.

(2) The bearing capacity and deformation capacity improved with the increase in the number of FRP layers. The increase in the number of FRP layers can also improve the second stiffness (i.e., the slope of the strengthening stage of the stress-strain curve) of the structure. Moreover, the concrete-filled CFRP-grooved steel composite tube columns show better bearing capacity, and the concrete-filled BFRP-grooved steel composite tube columns show better deformation capacity due to the better ductility of the BFRP.

(3) The stress-strain curves of the CFT and CFGT columns are composed of the initial linear part, elastic-plastic part and residual part, while the stress-strain curves of the CFGCT columns are composed of the initial linear part, the nonlinear transition part, the strengthening part and the residual part. In the initial linear part, the curves of the CFGT columns and the CFGCT columns are almost the same, while the slope of the initial stage of the CFT columns is larger due to the stress-released grooves. Then, after a nonlinear transition part when the steel tube yields, a strengthening part with a smaller slope appears in the curves of the CFGCT columns. The strengthening part is reflected only in the stress-overall strain curve of the CFGCT columns, which indicates that the FRP still provides a significant restraint effect after the steel tube yields. Finally, when the curve reaches the limit strain and the FRP breaks, the strain drops to a certain value and enters the residual part.

(4) Calculation formulas for the peak stress, peak strain, ultimate stress and ultimate strain were developed through a regression analysis of test data. Based on these formulas, a model for the stress-overall strain curves and stress-local strain curves of CFGCT columns was proposed. The proposed model can precisely simulate each stage of the stress-overall strain curve and stress-local strain curve of CFGCT columns and has excellent flexibility and versatility for practical application.

## Figures and Tables

**Table 1:** Test variables of all the specimens.

Specimens	Diameter *D* (mm)	Steel Thickness *t_s_* (mm)	Length *L* (mm)	Groove Width *t_g_* (mm)	Number of Grooves *n_g_*	Type	FRP Thickness *t_f_* (mm)	FRP Layers *n_f_*
S4.5N	133	4.5	400	5	4	-	-	0
S4.5B1	133	4.5	400	5	4	B	0.167	1
S4.5B2	133	4.5	400	5	4	B	0.334	2
S4.5B3	133	4.5	400	5	4	B	0.501	3
S4.5C1	133	4.5	400	5	4	C	0.167	1
S4.5C2	133	4.5	400	5	4	C	0.334	2
S4.5C3	133	4.5	400	5	4	C	0.501	3
S4.5NN	133	4.5	400	0	4	-	-	0
S6N	133	6	400	5	4	-	-	0
S6B1	133	6	400	5	4	B	0.167	1
S6B2	133	6	400	5	4	B	0.334	2
S6B3	133	6	400	5	4	B	0.501	3
S6C1	133	6	400	5	4	C	0.167	1
S6C2	133	6	400	5	4	C	0.334	2
S6C3	133	6	400	5	4	C	0.501	3
S6NN	133	6	400	0	4	-	-	0
S7.5N	133	7.5	400	5	4	-	-	0
S7.5B1	133	7.5	400	5	4	B	0.167	1
S7.5B2	133	7.5	400	5	4	B	0.334	2
S7.5B3	133	7.5	400	5	4	B	0.501	3
S7.5C1	133	7.5	400	5	4	C	0.167	1
S7.5C2	133	7.5	400	5	4	C	0.334	2
S7.5C3	133	7.5	400	5	4	C	0.501	3
S7.5NN	133	7.5	400	0	4	-	-	0

**Table 2:** Material properties of the FRP.

FRP Type	Nominal Thicknesses (mm)	Ultimate Tensile Strength (MPa)	Modulus of Elasticity (GPa)	Ultimate Strain
CFRP	0.167	4334.5	249.8	0.017
BFRP	0.167	1641.8	74.1	0.022

**Table 3:** Details of the test results.

Specimens	*f_lf_*	*f_ls_*	*ε_su_*	*P_max_*	*f_cc_*	*ε_cc,l_*	*ε_cc,a_*	*f_cu_*	*ε_cu,l_*	*ε_cu,a_*	*E_cs0,l_*	*E_cs0,a_*
	(MPa)	(MPa)		(kN)	(MPa)			(MPa)				
S4.5N	0	19.50	0.3339	1737.5	102.95	0.0016	0.015	125.12	0.0067	0.052	309,641	14,973
S4.5B1	4.12	19.50	0.3339	1721.6	106.53	0.0033	0.017	123.98	0.0071	0.046	265,201	14,924
S4.5B2	8.24	19.50	0.3339	1932.7	116.27	0.0029	0.023	139.18	0.0082	0.045	274,410	14,539
S4.5B3	12.36	19.50	0.3339	2047.1	117.06	0.0029	0.023	147.42	0.0094	0.048	258,962	14,991
S4.5C1	10.85	19.50	0.3339	1863.6	118.04	0.0032	0.023	134.21	0.0071	0.042	205,563	14,492
S4.5C2	21.71	19.50	0.3339	2127.5	122.16	0.0033	0.023	153.21	0.0079	0.041	248,423	15,297
S4.5C3	32.56	19.50	0.3339	2374.3	124.08	0.0030	0.028	170.99	0.0083	0.046	269,101	15,166
S4.5NN	0	20.61	0.3339	1584.3	91.5 0	0.0063	0.0063	131.30	0.0625	0.063	/	/
S6N	0	23.87	0.2882	1817.4	101.80	0.0015	0.016	130.88	0.0086	0.065	395,315	16,176
S6B1	4.12	23.87	0.2882	1787.2	108.21	0.0024	0.023	128.70	0.0104 *	0.047	365,055	16,186
S6B2	8.24	23.87	0.2882	1803.6	99.56	0.0022	0.020	129.89	0.0115	0.058	381,011	16,019
S6B3	12.36	23.87	0.2882	2207.8	114.94	0.0031	0.023	158.99	0.0133	0.051	348,733	14,173
S6C1	10.85	23.87	0.2882	1880.9	122.08	0.0016	0.028	135.45	0.0090 *	0.038	344,227	15,621
S6C2	21.71	23.87	0.2882	2307.0	129.43	0.0021	0.029	166.14	0.0071	0.046	332,994	15,716
S6C3	32.56	23.87	0.2882	2574.3	135.50	0.0029	0.031	185.39	0.0096	0.052	321,424	17,168
S6NN	0	25.25	0.2882	1697.2	98.10	0.0034	0.0034	147.70	0.0674	0.067	/	/
S7.5N	0	43.80	0.1444	2436.9	130.32	0.0022	0.026	175.49	0.0092	0.076	317,397	16,755
S7.5B1	4.12	43.80	0.1444	2487.8	122.68	0.0021	0.028	179.16	0.0103	0.057	305,209	15,377
S7.5B2	8.24	43.80	0.1444	2624.4	128.71	0.0024	0.026	189.00	0.0126 *	0.058	320,947	15,133
S7.5B3	12.36	43.80	0.1444	2670.0	135.14	0.0024	0.037	192.28	0.0135	0.060	326,212	18,116
S7.5C1	10.85	43.80	0.1444	2552.1	124.67	0.0022	0.032	183.79	0.0093	0.047	381,270	17,556
S7.5C2	21.71	43.80	0.1444	2707.8	130.90	0.0024	0.028	195.00	0.0095 *	0.050	389,680	17,093
S7.5C3	32.56	43.80	0.1444	3083.8	131.92	0.0025	0.026	222.08	0.0119 *	0.058	403,523	18,092
S7.5NN	0	46.40	0.1444	2522.5	143.30	0.0048	0.0048	222.90	0.0609	0.061	/	/

Notes: The local ultimate strain marked with * is obtained by extending the strain gauge test data. *f_cu_* is the ultimate stress; f*_cc_* is the peak stress; *ε_cu,l_* is the local ultimate strain; *ε_cc,l_* is the local peak strain; *ε_cu,a_* is the overall ultimate strain; *ε_cc,a_* is the overall peak strain; *f_lf_* is the lateral confining pressure of the FRP; *f_ls_* is the lateral confining pressure of the steel tube; *E_cs0,l_* is the initial slope of the stress-local strain curve; *E_cs0,a_* is the initial slope of the stress-overall strain curve.

**Table 4:** Summary of the database.

Researchers	Number	Diameter (mm)	*f_co_* (MPa)	FRP Type
Issa et al. [37]	10	150	55	CFRP
Chastre and Silva [38]	22	150	38	CFRP
Lee et al. [39]	23	150	36.2	CFRP
Eid et al. [40]	21	303	29.4–50.8	CFRP
Yin et al. [41]	18	150	30.6	CFRP
Huang et al. [42,43]	12	150	27.61/30.04	GFRP
This test	24	133	37.8	BFRP/CFRP

## Data Availability

The data presented in this study are available on request from the corresponding author.
